# Letter to the Editor: The effectiveness of early surgical stabilization for multiple rib fractures: a multicenter randomized controlled trial

**DOI:** 10.1186/s13019-023-02378-z

**Published:** 2024-01-03

**Authors:** Charlie Slowey

**Affiliations:** Department of Anesthesiology and Critical Care Medicine, Johns Hopkins Hospital, 21224 Baltimore, MD USA

To the Editor,

The author was pleased to read the latest addition to the evidence base for early surgical intervention of rib fractures presented by Wang et al. [1] We were surprised however that the authors did not include epidural analgesia incidence or effects of same in their paper. Epidural analgesia has mixed evidence regarding outcomes, in small randomized prospective trials it has been shown to reduce mechanical ventilation days [2] (one of Wang et al’s primary outcomes). However in larger retrospective analyses it would appear that epidural analgesia has no effect on mechanical ventilation time [3]. It is the author’s opinion that this study would benefit from a report on the incidence of patients who received epidural analgesia and the outcomes associated with its use in order to elucidate the potential benefits of epidural analgesia on chest wall injuries and to ensure no confounding is present.

Yours etc,

## Data Availability

Not applicable

## References

[1] Wang Z, Jia Y, Li M (2023). The effectiveness of early surgical stabilization for multiple rib fractures: a multicenter randomized controlled trial. J Cardiothorac Surg.

[2] Bulger EM, Edwards T, Klotz P, Jurkovich GJ (2004). Epidural analgesia improves outcome after multiple rib fractures. Surgery.

[3] Bachoumas K, Levrat A, Le Thuaut A, Rouleau S, Groyer S, Dupont H (2020). Epidural analgesia in ICU chest trauma patients with fractured ribs: retrospective study of pain control and intubation requirements. Ann Intensive Care.

